# A review of technical steps in the performance of arteriovenous fistula creation

**DOI:** 10.1177/11297298251328715

**Published:** 2025-04-24

**Authors:** Ben Edgar, Karen Stevenson, Emma Aitken, Andrew Jackson, Shannon Thomas, Maarten Snoeijs, Marco Franchin, Matteo Tozzi, David B Kingsmore

**Affiliations:** 1 School of Cardiovascular and Metabolic Health, University of Glasgow, Glasgow, UK; 2 Glasgow Renal and Transplant Unit, Queen Elizabeth University Hospital, Glasgow, UK; 3 Department of Surgery, Prince of Wales Hospital, Sydney, NSW, Australia; 4 Department of Vascular Surgery, Maastricht University Medical Center, Maastricht, The Netherlands; 5 Department of Surgery, University of Insubria, Varese, Italy; 6 Department of Vascular Surgery, Queen Elizabeth University Hospital, Glasgow, UK

**Keywords:** Renal dialysis, arteriovenous fistula, systematic review, randomised controlled trials as topic

## Abstract

Although it is accepted that a functional arteriovenous fistula (AVF) is the optimal vascular access for dialysis, achieving function is difficult as the outcomes of AVF creation are sub-optimal. Many technical steps have been proposed to improve outcomes, but the strength of evidence to support these is unclear. Thus, a systematic review of all randomised controlled trials (RCT) of operative strategies to optimise AVF outcomes was performed to summarise the evidence, review the overall level of standardisation in RCT and thus determine if there was an objective basis for the technical steps in AVF creation. A systematic review of all RCT was performed and studies categorised by intervention type. The rationale for each intervention, outcomes and limitations were described. Most importantly, the completeness of reporting procedural steps was compared for all RCT and the therapeutic impact considered by AVF site. Of 6741 records meeting the search criteria, 31 RCT were included. Most RCT did not control for all technical aspects or fully detail the operative methods, with a mean of 4 technical steps not reported for which other RCT have been performed. Of studies involving a surgical intervention in RCF, 10/13 reported a significant benefit compared to only 5/15 studies in BCF or larger vessels. Overall, the adequacy of reporting the technical details in all RCT of technical steps in AVF creation was poor. Despite this, there was a consistent patency benefit found in RCT performed in smaller vessels although the extent of interaction between these is uncertain. There remain gaps in the literature in defining the optimal steps in fistula creation that, if confirmed, could significantly improve AVF outcomes. This makes it essential that future studies of novel techniques, such as percutaneous AVF creation, incorporate a standardised operating procedure of optimal current practice of surgically created AVF as a meaningful comparator.

## Introduction

It is widely accepted and recommended in all guidelines that when established and functional, an arteriovenous fistula (AVF) is the optimal form of vascular access with the lowest complication rate and need for maintenance procedures. However, the outcomes for AVF creation are sub-optimal. Two meta-analyses reported primary patency rates of 64% at 1 year^1^ and 55% at 2 years.^2^ Longitudinal data shows that 61.9% of patients are re-admitted for vascular access procedures in the year following a primary operation.^3^ Furthermore, only 55% of AVF are used within 4 months of creation,^4^ and up to 70% of patients remain catheter dependent 8 months after AVF creation.^5^ Given these outcomes, it is unsurprising that industry has developed novel approaches to optimising outcomes and alternatives such as percutaneous AVF creation (pAVF).

The anastomosis of a small vein to a small artery has long been regarded as a demanding technical exercise with many variations and refinements proposed. The published evidence on technical steps of AVF creation is scattered across many widely differing journals, with differing study types and reviews that are selective and subjective.^6,7^ There has been no systematic review of the randomised literature focused solely on the technical steps of the surgical procedure, or the adequacy of describing this methodology. Without this, it is not possible to know how an ideal surgical AVF procedure (sAVF) should performed. Perhaps more importantly, determining what a standard sAVF procedure would entail is essential prior to performing an effective cost-efficacy focused RCT comparing alternative approaches such as pAVF.

Therefore, the aims of this review were: firstly, to summarise all the randomised evidence for RCT performed on the technical peri-operative surgical interventions undertaken to improve AVF outcomes; secondly, to assess the standardisation in RCT of technical steps other than the randomised step; thirdly, to determine if vessel size is an important factor in technique; fourthly, to compare these with the technical steps advocated for pAVF creation.

## Methods

This systematic review was registered on the PROSPERO international prospective register of systematic reviews (reference number CRD42024610219) and conducted in accordance with the Preferred Reporting Items for Systematic Reviews and Meta-Analyses (PRISMA) guidelines.

The search strategy aimed to identify any RCT that looked at one specific aspect of the surgical procedure. MEDLINE, Embase and Cochrane databases were systematically searched to identify all randomised controlled trials (RCT) published in the English that have reported on an intervention and AVF outcome. Reference lists of reviews, meta-analyses and reference lists were secondarily screened for literature not identified by the primary search strategies (Supplemental Material). Studies were included that described intra-operative decisions and interventions relating to the surgical site, anastomotic techniques and surgical modifications. Studies of experimental drugs, pre- and post-operative interventions or service provision (e.g. antiplatelets, exercise protocols, anaesthetic techniques, vein mapping, surveillance) were excluded.

Relevant outcomes included any description of fistula patency (primary, secondary, functional) at any time point. As this was an analysis of methodology of RCT, no meta-analysis was performed and thus the numbers and outcome terms reported in the original texts were reproduced irrespective of the adequacy and variation in outcome definition.

**Figure 1.**
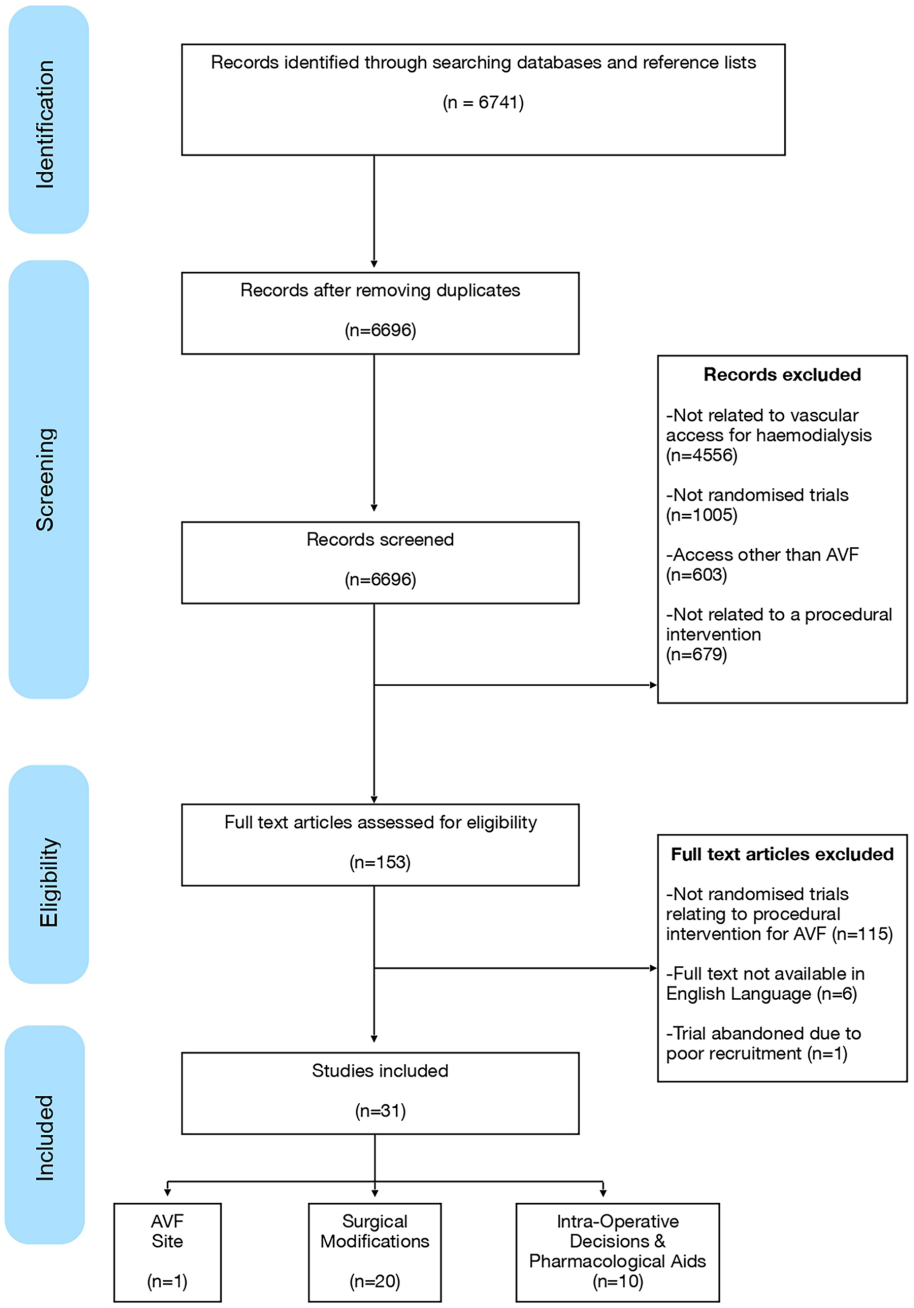
Identification and screening of included studies (PRISMA diagram).

Following removal of duplicates, titles and abstracts were screened for inclusion independently by two reviewers (BE, DBK) according to pre-specified criteria. Included titles proceeded to full text review (Figure 1). Data from these RCT included not only outcomes, but definitions and procedural steps.

RCT were categorised by the type of intraoperative intervention: AVF site, anastomotic techniques (suture technique, management of branches, vessel control, type of anastomosis, arteriotomy technique, dilation of vessels, anastomotic angle, atraumatic technique) and intra-operative drugs. Firstly, the rationale for the intervention was summarised with the design and outcomes of the RCT outlined. Secondly an analysis was performed of all RCT to look at the adequacy of the description of the methodology considering the steps studied in other RCT. Thirdly the impact of intervention was considered by AVF site (wrist vs upper arm). Finally, a comparison was made of what was effective in improving AVF outcomes in sAVF with pAVF protocols.

## Results

Thirty-one randomised controlled trials (RCTs) were identified and categorised as relating to the surgical site, anastomotic techniques and surgical modifications, or other intraoperative decisions and pharmacological aids (Figure 1).

### AVF site (1 RCT)

*Rationale*: The site chosen to create an AVF (hand, wrist, forearm or elbow) is a key initial decision, each having potential benefits: distal AVF have an optimal cannulation segment in the forearm and dual venous outflow at the elbow, reducing the risk of outflow stenosis.^8^ Rates of symptomatic central venous stenosis and steal syndrome are also lower in distal AVF,^9–11^ and there remains the option of a secondary more proximal AVF should the first fail. However observational data suggests that distal AVF have a lower patency rate.^12–14^ This may be due to several factors such as vessel size,^15^ relative ratio of vessel sizes^16^ and compliance or ability to dilate.^17,18^ Critically, observational data cannot determine whether the greater failure rate of distal AVF is solely because wrist vessels tend to be smaller, with more branches and of poorer quality from previous cannulation.

*Description of RCT*: A single RCT randomised patients suitable for an AVF creation at both sites to either wrist or elbow AVF (*n* = 230).^19^ A single surgeon performed all procedures under local anaesthetic. Patency was significantly lower in wrist AVF compared to elbow AVF (patency at 6-months: 74% vs 27%, *p* = 0.0039; 12-months: 66% vs 16.5%, *p* = 0.007). The rates of complications, technical details and peri-operative care were not reported.

### Anastomotic techniques + surgical modifications

#### Compliant suture line: (5 RCT)

*Rationale*: The original Carrel vascular anastomosis utilised an interrupted sutures technique (IS), however a continuous suture technique (CS) has become more widely used, perhaps as this is viewed as simpler and quicker. Other specialities performing microvascular anastomosis recommend IS with theoretical benefits that include a more physiological waveform, greater compliance and expansion over time with increased flow.^20,21^ In considering AVF creation, these benefits could improve AVF flow and thus patency but may also increase the risk of high-flow complications such as steal.

*Description of RCTs*: There are three methods described:

i.* Interrupted Sutures*: Interrupted sutures have been used as part of a technique^22^ and in special circumstances such as paediatric AVF creation.^23^ Only one RCT specifically randomised to IS versus CS (*n* = 78; single centre, multiple surgeons, RCF only).^24^ The primary patency was doubled with IS at 6 weeks (71% vs 47%, *p* = 0.01) with a non-significant improvement in functional patency (52% vs 36%, *p* = 0.18). The lack of significance may relate to the numbers achieving this end point being below the anticipated power calculation.
ii.* Continuous Sutures*: One RCT compared two continuous suturing techniques – parachute versus anchoring (*n* = 100, 94% proximal AVF).^25^ The use of a parachute technique saw a modest benefit in primary patency at 30 days (96% vs 80%, *p* = 0.014), but no significant difference in functional maturation at 6 weeks (85% vs 80%, *p* = 0.352).
iii.* Anastomotic Clips*: Three RCT have compared a clipped to a sutured anastomosis although these were all published more than 20 years ago and have not translated into widespread practice. An early RCT of a mixed cohort of all types of vascular access reported a benefit with clips in a sub-group analysis of AVF (*n* = 40 AVF; 2 year primary patency 71% vs 61%, *p* = 0.66; secondary patency 91% vs 61%, *p* = 0.05).^26^ A further small RCT found a non-significant benefit with clips (*n* = 31).^27^ The largest RCT (*n* = 107, all RCF) found no difference in PP, but a modest improvement with clips (mean secondary patency 435 vs 344 days, *p* = 0.009).^28^ However, in the authors experience, the widespread use of clips is not common.

#### Management of branches: (2 RCT)

*Rationale*: There are two options for managing branches of the main venous outflow at the time of AVF creation – ligation to increase flow in a single venous channel and improve maturation or preserving branches to reduce outflow resistance and increase the flow through the anastomosis to optimise early patency.

*Description of RCTs*: An RCT of preservation versus ligation of the distal vein in side-side snuffbox anastomosis (*n* = 60) found no differences in early or late outcomes.^29^ Similarly, an RCT of RCF (*n* = 115) randomised the management of the dorsal branch of the cephalic vein and found no significant difference in outcome (overall patency of 74% at 1 year).^30^

#### Anastomotic technique: Side-side (S-S) versus end-side (E-S): (3 RCT)

*Rationale*: There are two very differing philosophies of how to anastomose vessels. A side-side anastomosis may benefit from better flow characteristics and less surgical trauma in mobilising the vein than an end to side anastomosis. Three reviews/meta-analyses have been published, with none including all nine published RCT but all included a varying number of observational studies: 2018 – 7 studies (3 RCT)^31^; 2021 – 7 studies (5 RCT)^32^; 2022 – 16 studies (6 RCT).^33^ This may be explained by only three of the nine RCT being available in English.

*Description of RCTs*: A very early trial (*n* = 71, adults plus paediatric AVF) reported no patency differences at 9 months.^23^ A further RCT in an unknown population (*n* = 60) reported no differences in 6-month failure rate.^34^ The largest RCT (*n* = 100) randomised younger patients (mean age 38 years), creating both elbow and wrist AVF.^35^ The ‘functional maturation’ was better in the E-S group (35/50 vs 17/50, *p* = 0.0001), but there was no significant difference in 1-year secondary patency (84% vs 85%, *p* = 0.225). However, as the analysis of outcomes included all types of AVF, it remains uncertain if one technique is better or in what situation this is most clearly demonstrated.

#### Arteriotomy: (2 RCT)

*Rationale*: The method of creating an opening in an artery can be broadly categorised as either by a longitudinal slit or creating a hole. There is a functional difference between the two as a slit, which has a fixed length, relies on arterial compliance to increase the breadth of the lumen. Alternatively, an opening can be created by excising a disc of artery using scissors or an arterial punch, or by creating a flap in the artery. This then creates a lumen that is two dimensional with fixed length and breadth. The benefits of creating a fixed opening are a reduced likelihood of technical issues, and a guaranteed aperture even in non-compliant calcified arteries.

*Description of RCTs*: Two RCT have compared these techniques. One looked at a diamond or fish-mouth anastomosis compared to a slit (*n* = 56, any AVF, >2 mm vein) and reported better early patency, though functional outcomes were the same.^36^ A second RCT compared a v-flap technique to a slit in younger, fitter patients getting an RCF (*n* = 20).^22^ The v-flap technique resulted in increased flow across the anastomosis and shorter time to maturation (4 vs 8 weeks, *p* < 0.001). Interestingly, the V-flap technique also employed an interrupted suture technique.

#### Mechanical vessel dilation: (7 RCT)

*Rationale*: Blood vessels dilate and constrict in response to both physiological and non-physiological stimuli, such as surgical manipulation. Given that blood flow and vessel diameter are directly associated with outcomes, it is logical that any technique used to counteract surgically induced vasoconstriction may improve outcomes. Three methods have been proposed: hydrostatic dilatation (HD), mechanical probes (MD) and plain balloon angioplasty (PBA). Theoretically, HD dilates to a uniform pressure which can only be effective up to the level of the first collateral, is not measurable nor predetermined and is not uniform between cases. PBA dilates to a fixed diameter irrespective of pressure and thus may result in adverse venous trauma or even rupture. This pre-emptive PBA performed intra-operatively is an extension of PBA for non-maturing AVF which is safe and effective.^37^

*Description of RCTs*: Seven RCT and one meta-analysis of three RCT have been performed. The RCT varied in the selection criteria, adjuvant therapies and the techniques used.

i. *Dilation versus Non-dilation (4 RCT, 1 meta-analysis)*: PBA was compared to no dilation in a large RCT (*n* = 300, unselected AVF) with PBA performed to 1 mm greater than the preoperative US measurement.^38^ Despite the non-dilated group having a larger diameter of vein preoperatively, PBA decreased time to maturation (3.7 vs 5.9 weeks, *p* < 0.001) and increased functional maturation (93% vs 80%, *p* = 0.001), though with a higher complication rate (9.6% vs 5%).

Two RCT had randomised to HD or not exclusively in RCF. Following an initial RCT in rabbits, patients (*n* = 190, all RCF, mean diameter of artery and vein 2 mm) were randomised to HD or not.^39,40^ This is the only clinical study found with a supportive animal trial. The outcomes were excellent, with HD found to result in significantly better primary patency (97% vs 90% at 12 months, *p* < 0.05), although not secondary patency.

In contrast, a recent RCT (*n* = 54, all had heparin and aspirin, mean vein diameters 2.1 – 2.3 mm in each arm) found no differences in failure to mature rates, but a higher failure rate when HD was used (27% vs 11%, *p* = 0.012).^41^ The randomisation was of one step as part of a wider approach – the no-touch technique. However, the analysis of outcomes implying a poorer outcome from dilation included all outcomes (death, primary failure and failure to mature). A repeat analysis using the published data but excluding deaths, showed no significant difference (*p* = 0.136, authors analysis).

ii. *Type of Dilation (3 RCT)*: Two RCT comparing PBA versus HD have been performed in small veins (<2 mm diameter) with very similar outcomes. The first (*n* = 40; PBA to 4 mm) found early benefits in the PBA group (initial success 100% vs 67%, *p* = 0.04), and better 6-month primary patency (95% vs 57%, *p* = 0.01) although the ‘working rate’ was similar (100% vs 90%, *p* = 0.5).^42^ A further similar RCT (*n* = 60) found remarkably similar results: lower early failure with PBA (27% vs 0% *p* = 0.005), shorter time to maturation (52 vs 33 days, *p* < 0.001), lower 6-month re-intervention (37% vs 7%, *p* = 0.01), but both techniques had exceptionally good functional outcomes at 6 months (100% vs 90%, *p* = 0.23).^43^ A further RCT (*n* = 80) of BCF with small veins (<2.5 mm) found better outcomes with PBA, but none were statistically significant.^44^ However, there appears to be a misprint in the article as the numbers in the text, tables and abstract differ.

A systematic review + meta-analysis of three of the five RCT that included patients with veins <2.5 mm found PBA to be superior in all respects to HD. For example, the odds of 6-month primary patency were significantly higher following PBA (93.2% vs 64.5%; OR 6.09 (2.36–15.76), *p* = 0.0002).^45^ It is notable that the benefit of dilation, particularly PBA, is most clearly seen in primary rather than secondary patency. This could imply that PBA is not required in many cases, and that deferred dilation with PBA when required may be as successful as intra-operative PBA in every case.

A further different method of dilation using a malleable vascular dilator was compared to balloon ‘angioplasty’ (*n* = 120), with both methods being performed after initial hydrostatic dilation.^46^ However, the PBA described employed a short Fogarty catheter withdrawn through the vein rather than an angioplasty balloon which may explain the lack of benefits found at short or long-term follow up. Mechanical dilation has also been performed ad hoc in other RCT.^29,34^

#### Anastomotic angle: (1 RCT)

*Rationale*: Computational flow dynamics have been employed to study a potential relationship between vessel geometry and flow turbulence that may contribute to wall shear stress within the outflow vein and thus the risk of venous neointimal hyperplasia. Although studied for many years, no definitive link has been proven, although more recent approaches using detailed analysis of individual patients may help to understand the complex processes involved.^47,48^ Two devices have been commercially produced to enhance the haemodynamic flow (*Optiflow*^49^ *(Bioconnect Systems, Pennsylvania, USA)* and *VasQ (Laminate Medical Technologies, Israel)*), although only the *VasQ* device has been assessed in an RCT.^50^

*Description of RCT*: The *VasQ* device is an implantable external blood vessel support, regulating the anastomotic angle and creating a gentle radius of curvature which is hypothesised to improve flow dynamics and prevent venous stenosis. In an RCT (*n* = 40, all BCF) there was no significant difference in primary or secondary patency at 6 months (80% vs 66%, *p* = 0.5; 85% vs 77%, *p* = 0.6). Functional patency was improved with the device (100% vs 56%, *p* = 0.01) however this analysis excluded 17 out of the 40 recruited, presumably as they had not started dialysis.^50^ US detected stenosis was reduced from 50% to 15% (*p* = 0.04), but without a larger study with longer follow-up, it is unclear if the haemodynamic effects would translate into meaningful patency benefits.

#### Atraumatic technique (1 RCT)

*Rationale*: the ‘no-touch technique’ (NTT) aims to minimise trauma to the vessels by leaving the tissue surrounding the vessels intact, avoiding direct contact particularly with the vein, minimising the amount of flushing and avoiding direct manipulation of the vein.^51^

*Description of RCT*: An RCT compared NTT in RCF (*n* = 179, mean diameter artery and vein <2 mm) to a standardised procedure (both employing continuous suture, slit arteriotomy).^52^ A non-significant improvement in primary patency was found at 1 year (72% vs 62%, *p* = 0.138), but when restricted to small vessels (<1.8 mm veins), a significant benefit was found in primary patency (68% vs 40%, *p* = 0.004). The technique described emphasised maintaining the venous vascular pedicle. Other elements of NTT described in the original and observational studies were not clarified or differed for example, the use of systemic heparin, vasodilation, variation in anastomosis (S-S) and control of venous outflow.^53,54^

### Intra-operative drugs/pharmacological aids

#### Intraoperative drugs: (8 RCT)

*Rationale*: Intraoperative drugs may be delivered topically (e.g. papaverine) or systemically (e.g. heparin) and seek to promote vasodilatation and prevent early clot formation. Although heparin is established and used routinely in vascular surgery for anticoagulation, other significant non-anticoagulant effects are now recognised including dose-dependent vasodilation and an anti-inflammatory effect.^55,56^

*Description of RCTs*:

i. *Papaverine (1 RCT)*: An RCT (*n* = 110, all RCF, vein >3 mm) reported no difference in maturation rates, but a reduced maturation time with papaverine.^57^ Papaverine has also been used ad hoc in RCT of other interventions.^23,38^
ii. *Heparin (7 RCT)*: The use of heparin during AVF creation has been extensively studied and reviewed twice. An initial meta-analysis (3 RCT of AVF alone, 1 RCT of AVF and AVG) found that early patency rates in studies of AVF alone favoured heparin use (RR 0.57, 0.33–0.97, *p* = 0.04), but statistical significance was lost when AVG were also included. There was an overall significant increase in bleeding-related complications with heparin (RR 7.18, 2.41–21.38, *p* < 0.001).^58^ A more recent meta-analysis reviewed an additional three RCT (*n* = 7 in total).^59^ This came to similar patency findings (50% improvement in patency, RR 0.51, 0.31–0.85, *p* = 0.01) but found no increase in bleeding risk.^59^ There was recognition that the evidence available was generally of low quality due to variation in heparin dose, outcome measures and the case-mix of vascular access (exclusively RCF in some, any upper limb AVF in others, and also one that included AVG). A sub-analysis of two studies exclusively randomising RCF unfortunately omitted one study that was miss-classified and should have been included.^60^ An analysis of the remaining two studies showed the greatest benefits (early thrombosis: 0% vs 28%, *p* = 0.004,^61^ thrombosis at 4 weeks: 9% vs 27%, *p* = 0.03^62^) that translated to better maturation (‘presence of a thrill at 6 weeks’: 88% vs 60%, *p* = 0.024,^61^ failure to mature: 13.6% vs 31.8%, *p* = 0.040^62^). In contrast, the three RCT with the least benefit had mixed populations with up to 1/3 of the patients having an AVG in one study,^63^ or including BCF and BBF.^64,65^ This implies that a benefit from heparin reducing thrombosis may be most clearly seen in RCF.

#### Blood volume: (1 RCT)

*Rationale*: Thrombosis is more likely when blood flow is low, and thus increasing flow through the arteriovenous anastomosis is likely to improve early patency.

*Description of RCTs*: One RCT tested this hypothesis in RCF (*n* = 105, radial artery diameter ⩽1.6 mm).^66^ Patients were randomised according to receive either no additional fluid, or a plasma expander (an average of 720 ml of hydroxyethyl starch). This use of fluid improved immediate patency (‘technical success rate’ of 86% vs 26%, *p* < 0.001) which persisted on longer follow up (2-year patencies 66% vs 13%).

## The completeness of technical description

In the methodology of the RCT, 14 studies were noted to be single-surgeon, with 12 making no comment. The methodology of the RCTs analysed were compared for all technical aspects, which were chosen as these had been the subject of other RCT (Table 1). The entry criteria varied widely: AVF site – RCF only (*n* = 13), BCF only (*n* = 2), mixed cohorts (*n* = 14), Not described (*n* = 2). The details of the technical description of operative procedures varied widely. Of the RCT that randomised to a surgical modification (*n* = 28), a mean of 2.7 details were omitted (range 1–6). Given the importance of vessel size and site, studies were grouped by the inclusion criteria (RCF or small vessels vs mixed or BCF). In smaller vessels, 10/13 studies reported significant patency benefits to an intervention, whereas 5/10 studies in the larger vessels reported no benefits (Table 2).

**Table 1.** Reporting of technical considerations in trials of AVF interventions. Reporting of technical steps in RCT of AVF interventions.

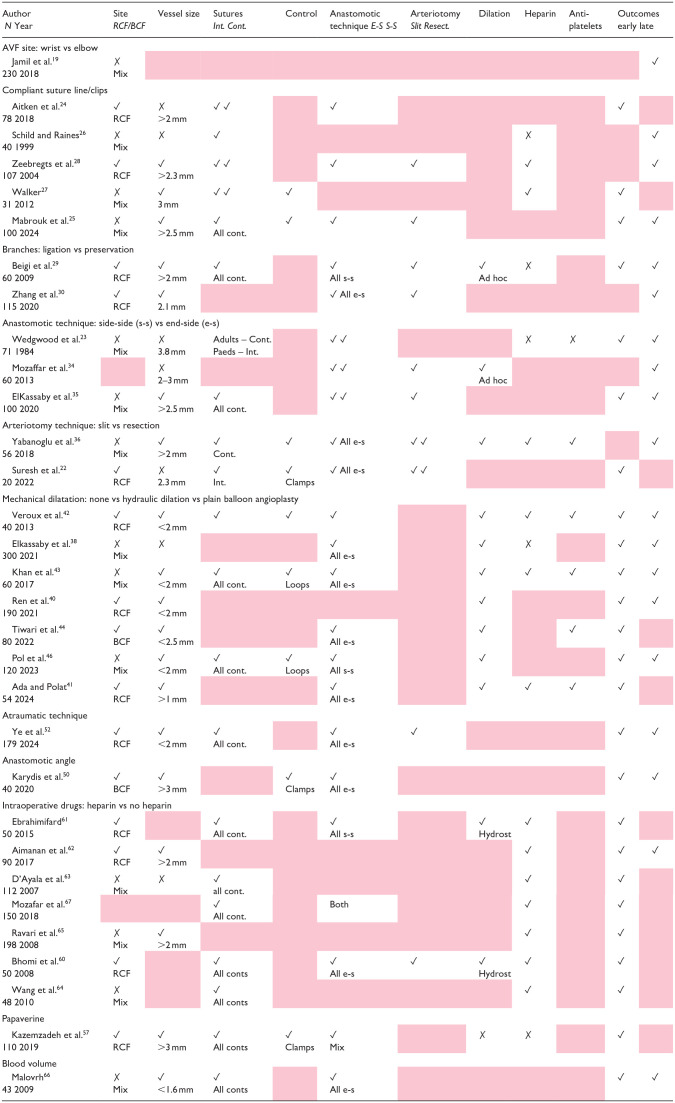											

RCF: radiocephalic fistula; BCF: brachiocephalic fistula; Mix: mixed population of RCF, BCF and AVG; Mix (AVF): mixed cohort but AVF analysed separately; Int.: interrupted; Cont.: continuous; Paeds: paediatric population; Hydrost: hydrostatic dilation; Loops: silicone vascular loops; E-S.: end to side; S-S.: side to side; Resect.: resectional.

✓ Kept uniform; ✗ Not kept uniform – but reported; gray: not reported.

**Table 2.** Patency benefits of surgical modifications grouped by trial inclusion criteria.

Author	Trial	Benefit	Outcome measure	Favoured	*p*-Value
Exclusively RCF or small vessels					
Aitken et al.^24^	Interrupted vs Continuous	Yes	Primary Patency	Interrupted	0.01
Zeebregts et al.^28^	Clips vs Sutures	Yes	Secondary Patency	Clips	0.009
Veroux et al.^42^	PBA vs HD	Yes	Primary Patency	PBA	0.01
Ren et al.^40^	HD vs nil	Yes	Primary Patency	Hydraulic dilation	<0.05
Ye et al.^52^	Min Trauma vs normal	Yes	Primary Patency	Atraumatic technique	0.0004
Khan et al.^43^	PBA vs HD	Yes	Primary Patency	PBA	0.01
Suresh et al.^22^	V-flap vs Slit Arteriotomy	Yes	Flow + maturation time	V-slit	0.001
Ebrahimifard^61^	Heparin vs nil	Yes	Functional Maturation	Thrombosis	0.004
Aimanan et al.^62^	Heparin vs nil	Yes	Functional Maturation	Thrombosis	0.04
Kazemzadeh et al.^57^	Papaverine vs nil	Yes	Maturation time	Papaverine	0.001
Pol et al.^46^	PBA vs Malleable Dilator	No	Improved patency both	–	–
Ada and Polat^41^	HD vs Not	No	Patency	–	–
Bhomi et al.^60^	Heparin vs nil	No	Functional Maturation	–	–
Mixed populations, or BCF					
Malovrh^66^	Plasma Expansion vs nil	Yes	2-Yr patency	Plasma Expansion	0.001
Elkassaby et al.^38^	Intraoperative PBA vs nil	Yes	Functional Maturation	PBA	0.001
Karydis et al.^50^	VasQ vs nil	Yes	*Functional Patency	VasQ	0.01
Mabrouk et al.^25^	Anchor vs Parachute suture	Yes	Primary Patency	Parachute	0.014
Schild and Raines^26^	Clips vs Sutures	Yes	Secondary Patency	Clips	0.05
Tiwari et al.^44^	PBA vs HE	No	All outcomes	–	–
ElKassaby et al.^35^	End-Side vs Side-Side	No	Secondary Patency	–	–
Mozaffar et al.^34^	End-Side vs Side-Side	No	Functional Patency	–	–
Pol et al.^46^	Intraoperative PBA vs nil	No	Functional Maturation	–	–
D’Ayala et al.^63^	Heparin vs nil	No	Patency	–	–
Ravari et al.^65^	Heparin vs nil	No	Patency	–	–
Wang et al.^64^	Heparin vs nil	No	30-day patency	–	–
Walker^27^	Clips vs Sutures	No	–	–	–
Yabanoglu et al.^36^	Diamond vs Slit	No	Functional Patency	–	–
Wedgwood et al.^23^	End-Side vs Side-Side	No	–	–	–

nr: not reported; HE: hydraulic expansion; PBA: Plain Balloon Angioplasty.

Excluded: Zhang, Beigi – branch ligation

*Analysis limited, presumably to patients who were on dialysis at 6 months.

## Discussion

The anastomosis of a peripheral vein to an artery has long been regarded as a demanding technical exercise in surgical specialities such as plastic, cardiothoracic and vascular surgery. Thus, it is unsurprising that alternative technical steps have been studied to try and improve results. This review aimed to examine the randomised literature on the procedural steps in creating an AVF to determine if there was evidence for an ‘ideal’ technical procedure. Although there are 31 RCT that have randomised one aspect of the surgical procedure, there are significant limitations in the methodology of all RCT. These limitations are three-fold: firstly, in not limiting the analysis by AVF site, secondly, in not standardising all other steps in AVF procedures other than the randomised step; thirdly, in not using standard definitions of endpoints or longer-term follow-up.

*AVF site*: The initial choice of site of an AVF is a key decision in clinical practice and in research studies. One RCT supports observational data showing that distal AVF (RCF) have poorer outcomes than proximal AVF (BCF), irrespective of size.^19^ Thus, RCT performed to evaluate patency in BCF or mixed populations will require significantly larger sample sizes or risk non-significant outcomes due to a Type II error. This may explain why there are many RCT that indicate a technical step to improve patency in RCF, but few have been substantiated in BCF other than intraoperative balloon angioplasty and potentially the VasQ device. Alternatively, optimising the technical steps may be critical in determining RCF patency; but less so for BCF in which outcomes other than patency, such as steal or high flow, may be more relevant. These are rarely reported.

*Methodology of RCT*: This review was principally aimed at an analysis of methodological quality of the RCT on technical aspects of AVF creation. This was based on whether key steps were reported. These key steps were chosen as those for which other RCT had been performed for example, suture technique, anastomosis creation, arteriotomy, dilation techniques, use of heparin. Whilst the evidence for each of these steps is weak, it is important to ensure that both the control arm and the interventional arm were replicated in all aspects other than the randomised intervention. No RCT in this analysis was complete in recording all technical steps. Many of the studies (14/31) were performed by a single surgeon and thus likely to have repeatable techniques with minimal variation. However, it makes it difficult to determine the value of the RCT when the other technical aspects are unknown.

*Endpoints*: Fundamentally, in determining the benefit of a technical RCT, it is crucial to determine firstly the most appropriate endpoint to judge success, and secondly the population in which to measure this endpoint. This review, similar to previous meta-analyses, was hindered by vague and ill-defined, subjective outcome reporting. The definition of appropriate clinical trial endpoints in vascular access is complex, with differing definitions of success and varied adverse outcomes that may be specific to the type of access created.^9,68^

Overall, the technical adequacy of detail of the procedural steps in the RCT analysed was poor with many not detailing potentially critical details in the performance of AVF surgery. Given the vast, complex array of influencing factors, it is not surprising that RCT are poorly standardised and not commonly performed. Unsurprisingly the literature base consists of only a few RCT for every potential intervention, yet a great number of large observational reports with uncertain selection bias. Meta-analyses may miss RCT, assess heterogenous outcome measures, be unable to use original data and do not consider the procedural details of the studies reviewed.

There remain large gaps in the literature regarding the optimal steps in fistula creation in which no RCT has been attempted. For instance, the method by which vessels are controlled may influence outcomes due to induced vascular spasm or direct arterial damage. Several methods of control have been described (microvascular bulldog clamps, silicone vascular loops, tourniquet,^69,70^ external manual compression, mechanical intraluminal probes/balloons^34,71,72^), but to date no RCT have been performed, despite some surgeons considering this a key way to improve outcomes.

The critical interpretation of this review lies in the lack of reproducibility of the trials described. Whilst interventions such as anastomotic clips, primary balloon angioplasty and no-touch techniques have been associated with improved outcomes, each trial failed to report technical considerations which would be vital to replicate their technique (Table 1). For example, vein dilatation was variably used in some RCT, but not on a routine basis.^29,34^ As it was not the intervention under scrutiny and no sub-analysis performed based on whether it was performed, it is impossible to determine how it may have affected outcomes.

Despite the limitations, there is broad evidence that improvements in the outcomes of distal AVF are attainable and highlights the need for future studies to adhere to standardised operating procedures with adequate detail such that a true ‘gold-standard’ in surgical AVF creation might be defined. This is increasingly important as novel techniques are introduced into practice. Minimally invasive percutaneous AVF (pAVF) creation devices have been widely publicised in the last 5 years with good immediate outcomes. Protocols for technical performance highlight several key steps including very detailed pre-operative vein mapping often by the interventionalist, vessel dilation, peri-procedural heparin, post-procedure dual antiplatelets and secondary dilation procedures. This review has shown that there is RCT evidence for many of these steps in the performance of standard AVF creation. It would be interesting to know if there was any benefit of pAVF over standard AVF creation if both were performed to same optimal level of attention to procedural steps with randomised evidence, experience and detailed protocols.

## Supplemental Material

sj-pdf-1-jva-10.1177_11297298251328715 – Supplemental material for A review of technical steps in the performance of arteriovenous fistula creation Supplemental material, sj-pdf-1-jva-10.1177_11297298251328715 for A review of technical steps in the performance of arteriovenous fistula creation by Ben Edgar, Karen Stevenson, Emma Aitken, Andrew Jackson, Shannon Thomas, Maarten Snoeijs, Marco Franchin, Matteo Tozzi and David B Kingsmore in The Journal of Vascular Access
