# Supplementary material for: A review of technical steps in the performance of arteriovenous fistula creation
Source: J Vasc Access. 2025 Apr 24;27(1):90–101. doi: 10.1177/11297298251328715 (PMC12812182; doi:10.1177/11297298251328715)
Supplement: sj-pdf-1-jva-10.1177_11297298251328715 – Supplemental material for A review of technical steps in the performance of arteriovenous fistula creation [file sj-pdf-1-jva-10.1177_11297298251328715.pdf]

Ovid MEDLINE(R) ALL <1946 to December 31, 2024>
Embase 1947-Present, updated daily

1	random*.mp. [mp=ti, bt, ab, ot, nm, hw, fx, kf, ox, px, rx, ui, sy, ux, mx, tn, dm, mf, dv, dq]
2	trial.mp. [mp=ti, bt, ab, ot, nm, hw, fx, kf, ox, px, rx, ui, sy, ux, mx, tn, dm, mf, dv, dq]
3	RCT.mp. [mp=ti, bt, ab, ot, nm, hw, fx, kf, ox, px, rx, ui, sy, ux, mx, tn, dm, mf, dv, dq]
4	intervention*.mp. [mp=ti, bt, ab, ot, nm, hw, fx, kf, ox, px, rx, ui, sy, ux, mx, tn, dm, mf, dv, dq]
5	1 or 2 or 3 or 4
6	fistula.mp. [mp=ti, bt, ab, ot, nm, hw, fx, kf, ox, px, rx, ui, sy, ux, mx, tn, dm, mf, dv, dq]
7	AVF.mp. [mp=ti, bt, ab, ot, nm, hw, fx, kf, ox, px, rx, ui, sy, ux, mx, tn, dm, mf, dv, dq]
8	exp arteriovenous shunt, surgical/
9	vascular access.mp. [mp=ti, bt, ab, ot, nm, hw, fx, kf, ox, px, rx, ui, sy, ux, mx, tn, dm, mf, dv, dq]
10	(dialysis adj3 access).mp. [mp=ti, bt, ab, ot, nm, hw, fx, kf, ox, px, rx, ui, sy, ux, mx, tn, dm, mf, dv, dq]
11	(haemodialysis adj3 access).mp. [mp=ti, bt, ab, ot, nm, hw, fx, kf, ox, px, rx, ui, sy, ux, mx, tn, dm, mf, dv, dq]
12	AV fistula.mp. [mp=ti, bt, ab, ot, nm, hw, fx, kf, ox, px, rx, ui, sy, ux, mx, tn, dm, mf, dv, dq]
13	arteriovenous fistula.mp. [mp=ti, bt, ab, ot, nm, hw, fx, kf, ox, px, rx, ui, sy, ux, mx, tn, dm, mf, dv, dq]
14	6 or 7 or 8 or 9 or 10 or 11 or 12 or 13
15	5 and 14
16	exp animals/ not humans.sh.
17	15 not 16
18	limit 17 to english language
19	limit 18 to full text

Cochrane Library

1	MeSH descriptor: [Randomized Controlled Trial] explode all trees
2	MeSH descriptor: [Clinical Trial] explode all trees
3	MeSH descriptor: [Controlled Clinical Trial] explode all trees
4	((random*) near/3 trial):ti,ab,kw
5	#1 or #2 or #3 or #4
6	MeSH descriptor: [Vascular Surgical Procedures] explode all trees
7	MeSH descriptor: [Arteriovenous Fistula] explode all trees
8	#6 or #7
9	#5 and #8
10	avf:ti,ab,kw
11	fistula:ti,ab,kw
12	(“vascular access”):ti,ab,kw
13	("dialysis" or "hemodialysis" or "haemodialysis") near/3 access
14	#10 or #11 or #12 or #13
15	#9 and #14

(word variations searched throughout)
